# Comparison of the efficiency of different cell lysis methods and different commercial methods for RNA extraction from *Candida albicans* stored in RNAlater

**DOI:** 10.1186/s12866-019-1473-z

**Published:** 2019-05-14

**Authors:** Antonio Rodríguez, Mario Vaneechoutte

**Affiliations:** Laboratory Bacteriology Research, Department of Diagnostic Sciences, Faculty of Medicine and Health Sciences, Ghent University, C. Heymanslaan 10, 9000 Ghent, Belgium

**Keywords:** RNA extraction, Cell lysis, *Candida albicans*

## Abstract

**Background:**

Obtaining sufficient RNA yield and quality for comprehensive transcriptomic studies is cumbersome for clinical samples in which RNA from the pathogen is present in low numbers relative to the nucleic acids from the host, especially for pathogens, such as yeasts, with a solid cell wall. Therefore, yeast cell lysis including cell wall disruption constitutes an essential first step to maximize RNA yield. Moreover, during the last years, different methods for RNA extraction from yeasts have been developed, ranging from classic hot phenol methods to commercially available specific kits. They offer different RNA yield and quality, also depending on the original storage medium, such as RNAlater.

**Results:**

We observed that, for *C. albicans* cells stored in Tryptic Soy Broth with 15% glycerol, 10 min of bead beating in a horizontal position in RiboPure Lysis Buffer provided complete cell lysis. Cell lysis efficiency was decreased to 73.5% when cells were stored in RNAlater. In addition, the RiboPure Yeast Kit (Ambion) offered the highest RNA yield in comparison with the automated platform NucliSENS easyMAG total nucleic extraction (bioMérieux) and the RNeasy Mini Kit (Qiagen) according to NanoDrop and Fragment Analyzer. Moreover, we showed that, in spite of the decrease of cell lysis efficiency after RNAlater storage, as compared to storage in TSB + 15% glycerol, RNAlater increased RNA yield during RNA extraction with both RiboPure Yeast Kit and easyMAG, as confirmed by Fragment Analyzer analysis and by RT-qPCR of the RNA from the Internal Transcribed Spacer 2.

**Conclusions:**

In our hands, the most efficient cell lysis and highest RNA yield from *C. albicans* cells stored in RNAlater was obtained by horizontal bead beating in RiboPure Lysis Buffer followed by RNA extraction with the RiboPure Yeast Kit.

**Electronic supplementary material:**

The online version of this article (10.1186/s12866-019-1473-z) contains supplementary material, which is available to authorized users.

## Background

*Candida albicans* is the main infectious agent responsible for oral and vaginal candidiasis [1]. The fungus is usually a harmless inhabitant of the mucosal surfaces, but the loss of local host defense mechanisms together with the intrinsic virulence factors of *Candida* spp. frequently disrupt this co-existence [2]. Occasionally, the fungus can reach the bloodstream and the infection turns into a systemic disease, becoming fatal in immuno-compromised individuals [3]. The need for faster diagnostic tools in life-threatened patients, the high rate of recurrence in which patients suffer new episodes of candidiasis after anti-fungal therapy and the continued development of Next-Generation Sequencing techniques have boosted the study of host-pathogen interactions. Several reports have focused on transcriptomic analysis for a better understanding of the genes involved in these host-pathogen interactions [4, 5]. Although much has been elucidated about the human response, better understanding of the expression of fungal genes, which are in a very low proportion as compared to the human counterpart, is still lacking. There have been different attempts to resolve this question. The use of *Candida*-specific probes, that hybridize with all mRNAs of *Candida*, including the different splicing variants, to enrich fungal RNA is a promising method to improve our knowledge on this topic [6]. In addition, RNA extraction improvements in order to obtain high RNA yield from yeasts may be a way forward. There are many reports comparing different methods for RNA extraction in different microorganisms such as viruses [7, 8], bacteria [9, 10], and also fungi [11, 12]. The latter require additional cell wall disruption, and therefore more complex cell lysis approaches. Cell wall disruption methods are mainly focused on bead beating and enzymatic treatment and their efficiency has been shown to vary even with cell cycle [13]. Whereas most studies on Fungi deal with *Saccharomyces cerevisiae*, methods for maximal cell lysis efficiency, a crucial step prior to RNA extraction, and for RNA extraction, have not been reported in *C. albicans* so far. Cell lysis itself is not only important to increase yields of nucleic acids (incl. RNA), but also for other downstream applications requiring cell lysis such as those involving proteins such as SDS-PAGE, western blot, ELISA, MALDI-TOF or MS/MS.

In this study, we compared different cell lysis methods and evaluated cell lysis efficiency through microscopic visualization. In addition, we compared three commercially available RNA extraction methods and determined RNA yield, RNA purity and RNA integrity. In addition, we studied the effect of storage of cells in RNAlater on the efficiency of cell lysis and RNA extraction. Finally, we assessed amplifiability of the obtained RNA by means of reverse transcription and amplification (RT-qPCR) of the Internal Transcribed Spacer 2 (ITS2) RNA of the obtained RNA-extracts.

## Results

### Brief outline of the study

**Fig. 1:**
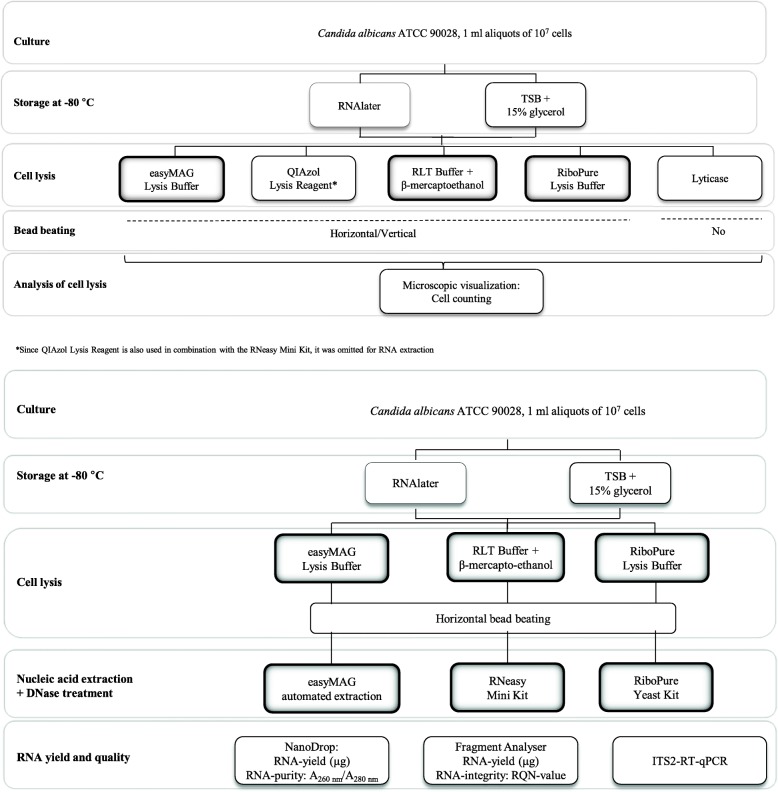
A schematic overview of the study set up for **a**) cell lysis and **b**) RNA extraction

To evaluate which of different cell lysis methods and commercial RNA extraction kits were the most efficient to obtain high yield and high quality RNA from yeast cells, we prepared 1-ml aliquots, each containing 10^7^ *C. albicans* cells in log growth phase, and stored these at − 80 °C in RNAlater vs Tryptic Soy Broth + 15% Glycerol (TSB-G, as a control). We compared lysis of thawed aliquots with Lyticase in Lyticase Lysis Buffer (LLB) without bead beating - as the control method proposed to yield very efficient lysis - vs bead beating in four different lysis buffers. We also carried out bead beating in two different vortexes, a normal vortex in which tubes are oriented vertically and a hands-free vortex that allows bead beating in a horizontal position and thus increases the lysis area. Subsequently, we evaluated microscopically the percentage of cell lysis that could be obtained with the different treatments. Finally, we applied three commercially available RNA-extraction methods that were used after cell lysis with their cognate cell lysis method and determined RNA-yield (Fragment Analyzer and NanoDrop), RNA-purity (NanoDrop), RNA-integrity (Fragment Analyzer) and efficiency of reverse transcription and amplification (RT-qPCR) of the ITS2 RNA of the obtained RNA-extracts. A general outline of the study is depicted in Fig. 1a and Fig. 1b.

### Bead beating combined with RiboPure lysis buffer yields maximum cell lysis

**Fig. 2:**
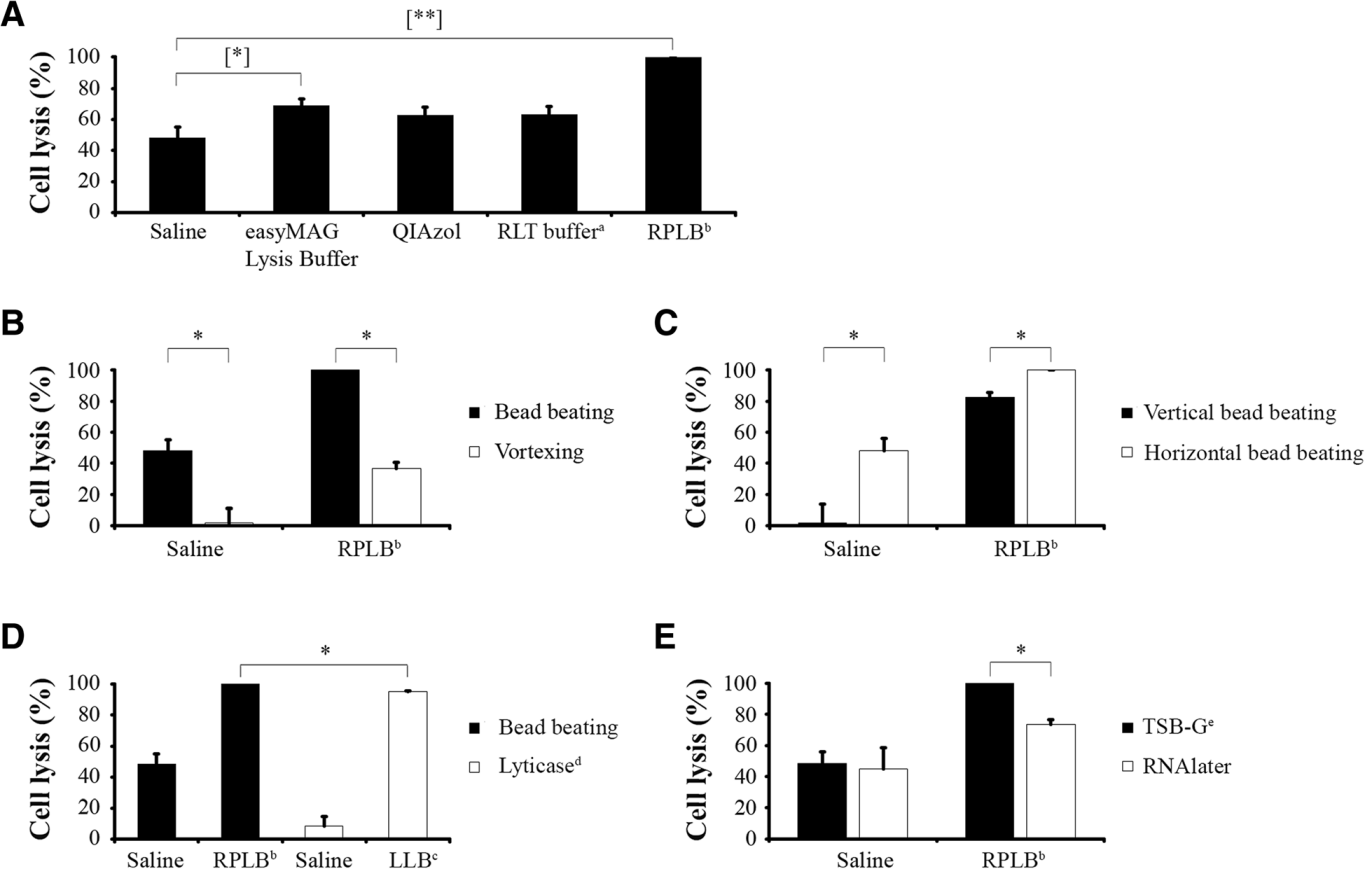
Percentage of cell lysis obtained after different treatments. **a** Bead beating during 10 min in a horizontal position. **b** Horizontal bead beating and vortexing (without beads). **c** Bead beating in vertical and horizontal position. **d** Horizontal bead beating and lyticase treatment. **e** Bead beating in horizontal position after different storage conditions Error bars represent the standard deviations of results from six biological replicates. Statistically significant differences are marked with an asterisk (*P* < 0.05) (pair samples Wilcoxon test) and with one (*P* < 0.05) or two (*P* < 0.005) bracketed asterisks (Friedman test). **a**: RLT buffer (RNeasy Mini Kit) + 1% *β*-mercaptoethanol. **b**: RiboPure Lysis Buffer. **c**: Lyticase Lysis Buffer. **d**: 2 U/μl lyticase for 1 h. **e**: Tryptic Soy Broth + 15% glycerol

Cell lysis is a crucial step during RNA extraction since the amount of lysed cells impacts directly on the amount of RNA obtained at the end of the process. Here, we tested cell lysis efficiency by using four different lysis buffers, i.e., easyMAG Lysis Buffer, RLT buffer (RNeasy Mini Kit) + 1% *β*-mercaptoethanol (i.e., 143 mM), QIAzol Lysis Reagent and RiboPure Lysis Buffer (RPLB), which were all used in combination with 10 min of horizontal bead beating with 0.5 mm zirconium beads in a vortex fitted with a vortex adapter. This adapter allows hands-free vortexing and also bead beating in a horizontal position, increasing the surface area for shearing and lysis. Using microscopy, we observed 100% cell lysis when *Candida* cells, stored in TSB-G, were bead beaten with RPLB, which outperformed all other lysis methods tested, since easyMAG, QIAzol and RLT buffers resulted in 60 to 70% of cells lysed (Fig. 2a) and the saline control resulted in 48% cell lysis. In addition, we tried to quantify cell lysis by culture of cells on blood agar plates, but growth was impaired because of the inhibition effect of all of the lysis buffers, as we observed that a concentration of 1% lysis buffer already resulted in absence of growth (data not shown). In conclusion, in our hands, bead beating during 10 min in RPLB was shown to be the most efficient method to lyse yeast cells.

### Bead beating vs vortexing without beads in RPLB and saline

As we obtained 100% cell lysis of *C. albicans* cells stored in TSB-G by means of horizontal bead beating in RPLB, we asked whether vortexing itself, without beads, would be sufficient to lyse yeast cells. Horizontal bead beating and vortexing without beads were compared, resulting in 37% of cell lysis when vortexing in the absence of beads, as shown in Fig. 2b. Thus, cell lysis was significantly reduced in RPLB when beads were removed (*p* < 0.05). We concluded that vortexing alone is not sufficient and that bead beating is needed to lyse yeast cells properly.

### Horizontal vs vertical position bead beating

As we observed that bead beating in a horizontal position was an essential step for the most effective lysis of yeast cells (see *Bead beating* vs *vortexing without beads in RPLB and saline*), we wondered about the importance of the horizontal orientation of the tubes for the lysis of *Candida* cells. To test this, we compared horizontal bead beating with an adapter during 10 min, as described above, with manual vortexing in a vortex mixer in which tubes are bead beaten manually in a vertical position during 10 min, and we did so only for the RPLB and only after storage in TSB-G.

Comparing horizontal vs vertical bead beating, we observed an average of 48% vs 2% cell lysis respectively in saline and 100% vs 83% respectively in RPLB, each for six different replicates (Fig. 2c). These differences were statistically significant (*p* < 0.05), and indicate that horizontal bead beating with a vortex adapter is more efficient than vertical bead beating.

### Bead beating vs Lyticase treatment

After having shown that horizontal bead beating was more efficient than vertical bead beating, that bead beating was more efficient than vortexing alone and that RPLB was the most efficient lysis buffer, we next asked whether enzymatic treatment with lyticase would be more efficient than bead beating to lyse yeast cells. For this purpose, we incubated the yeast cells in LLB containing 2 U/μl lyticase during 1 h, a standard lysis procedure for yeast cells that e.g. is being used in a commercially available protocol (DNA extraction kit, Qiagen) and that was used in our previous studies [14]. Lyticase treatment was shown to be very efficient with 95% cell lysis (Fig. 2d). However, horizontal bead beating with RPLB significantly lysed more cells (100%) than lyticase treatment (*p* < 0.05).

### Cell lysis is affected by RNAlater

RNAlater is an RNA protective agent that has been shown to perform equally well as snap-frozen methods to stabilize transcriptomic profiles [15]. In addition, it has already been used in clinical samples to study the transcriptomic profile of *C. albicans* [5]. However, the effect of RNAlater on cell lysis efficiency has not been tested yet. Therefore, we checked whether cell lysis was influenced when samples are stored in RNAlater as compared to samples stored in TSB-G, and we compared cell lysis efficiency after horizontal bead beating in RPLB, for both storage methods.

For cells stored in RNAlater, we observed a cell lysis efficiency of 73.5%, when horizontal bead beating was carried out in RPLB, compared to cells stored in TSB-G (100%) (Fig. 2e).

No such reduction of cell lysis efficiency was observed when horizontal bead beating was carried out in saline (instead of RPLB) in which 44.7 and 48.3% cell lysis was achieved after storage in RNAlater or in TSB-G, respectively. Thus, these results indicate that RNAlater is impairing cell lysis, but only when horizontal bead beating is carried out in RPLB.

### The RiboPure yeast kit provides the highest RNA yield

**Fig. 3:**
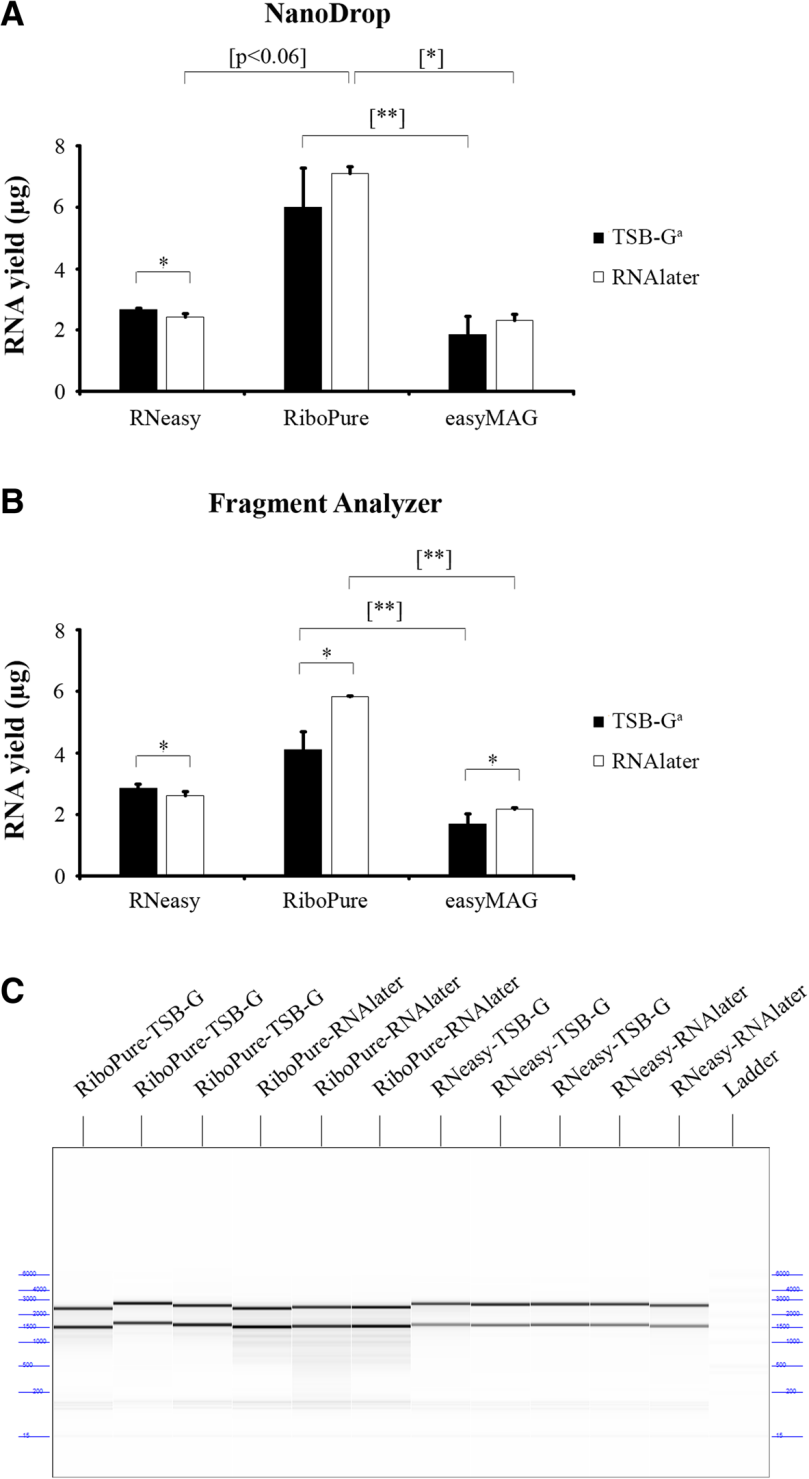
Comparison of RNA yield and quality for three different RNA-extraction methods. RNA yield from 10^7^ *Candida* cells was measured with both **a**) NanoDrop spectrophotometer (Isogen) and **b**) Fragment Analyzer (Advanced Analytical Technologies). **c** RNA quality of some of the samples is shown in a gel image. A minor shift in size is observed among rRNA bands. According to the manufacturer, this has been observed before and might be explained as possibly related to the expiration of the polymer (ThermoScientific. Fragment Analysis Support–Troubleshooting). Error bars represent the standard deviations of results from six biological replicates. Statistically significant differences are marked with an asterisk (*P* < 0.05) (pair samples Wilcoxon test) and with one (*P* < 0.05) or two (*P* < 0.005) bracketed asterisks (Friedman test). **a**: Tryptic Soy Broth + 15% glycerol

After optimization of the cell lysis process, we evaluated whether the differences observed during cell lysis among different lysis buffers translated into differences in RNA yield and RNA purity after RNA extraction with the corresponding extraction kit. To that purpose, we compared three commercially available methods for nucleic acid extraction of *Candida* cells: the NucliSENS easyMAG, the RNeasy Mini Kit and the RiboPure Yeast Kit, following horizontal bead beating in their cognate lysis buffers. These nucleic acid extractions were followed by DNase treatment to degrade chromosomal DNA. Yield was determined with NanoDrop and Fragment Analyzer. The RiboPure Yeast Kit produced 7 and 6 μg of RNA from yeast cells stored in RNAlater and TSB-G, respectively, according to NanoDrop (Fig. 3a). The yields obtained with the RiboPure Yeast Kit were significantly higher than those obtained with the RNeasy Mini Kit (2.5 and 2.8 μg) and the easyMAG (2.0 and 1.0 μg). Comparable results were obtained after quantification with Fragment Analyzer (Fig. 3b). RNA yield after extraction with the RNeasy Mini Kit was significantly lower in samples pre-stored in RNAlater in comparison with samples stored in TSB-G, according to both NanoDrop and Fragment Analyzer. However, RNAlater significantly increased RNA yield with both the easyMAG and the RiboPure Yeast Kit according to Fragment Analyzer. This increment was also appreciated with NanoDrop, but did not reach significance, because of a larger standard error. It can be concluded that the RiboPure Yeast Kit provides the highest RNA yield and that RNAlater further improved RNA extraction with easyMAG and RiboPure Yeast Kit.

Besides RNA yield we also assessed RNA quality through RNA integrity and purity, because both parameters are taken into account in most RNA-based methods such as RNAseq. Table 1 shows RNA yield (μg), RNA purity (A260/A280 ratio) and RNA integrity (RQN value) obtained for the three RNA extraction methods.

**Table 1:** RNA yield and quality for three different RNA extraction methods^a^

Storage medium	RNA extraction method	RNA yield and quality^b^			
		NanoDrop		Fragment Analyzer	
		RNA yield (μg)	A260/A280^c^	RNA yield (μg)	RQN^d^
TSB-G^e^	RNeasy Mini Kit	2.68 ± 0.04	1.93 ± 0.04	2.87 ± 0.13	10.00 ± 0.00
TSB-G^e^	RiboPure Yeast Kit	6.01 ± 1.27	2.02 ± 0.03	4.12 ± 0.57	9.50 ± 0.02
TSB-G^e^	NucliSENS easyMAG	1.86 ± 0.59	2.14 ± 0.08	1.70 ± 0.32	9.87 ± 0.12
RNAlater	RNeasy Mini Kit	2.43 ± 0.11	1.84 ± 0.06	2.61 ± 0.15	10.00 ± 0.00
RNAlater	RiboPure Yeast Kit	7.10 ± 0.22	1.98 ± 0.02	5.82 ± 0.03	7.77 ± 0.75
RNAlater	NucliSENS easyMAG	2.32 ± 0.20	2.01 ± 0.07	2.17 ± 0.06	10.00 ± 0.00

^a^All extraction methods after horizontal bead beating in cognate lysis buffer

^b^Results are means and standard deviations of six independent extractions

^c^Quality for NanoDrop is shown as the A260/A280 ratio. A value of ~ 2.0 is generally accepted as indicating that RNA is free of proteins

^d^Quality for Fragment Analyzer is shown as the RQN value. Reported on a scale of 1 to 10, with higher values indicating a better quality of total RNA. Values above 7 are considered to represent high quality and non-degraded RNA

^e^Tryptic Soy Broth + 15% glycerol

We observed A260/A280 ratios close to 2.0 for all three methods, including for samples pre-stored in RNAlater, except for the RNeasy Mini Kit in which RNA purity was significantly lower (*p* < 0.05) after RNAlater storage. RQN values were also excellent, i.e., around 10 for all of the methods, except for the RiboPure Yeast Kit after RNAlater storage, i.e., ~ 8 which was significantly lower (*p* < 0.05) than after storage in TSB-G, i.e., 9.5. More detailed electropherograms (Additional file 1: Figure S1) and gel images of some samples (Fig. 3c) are shown. In general, these results indicate that all RNA extraction methods provide high RNA quality and yield and that they can be used in combination with RNAlater storage.

### Specific RT-qPCR confirmed the RiboPure yeast kit as the most efficient RNA extraction

To further validate the results obtained after RNA extraction, quantitative reverse transcription PCR (RT-qPCR) was carried out, amplifying the ITS2 RNA. RNA was extracted from 10^7^ yeast cells, pre-stored in RNAlater or TSB-G, with the three commercially available RNA extraction methods (easyMAG, RNeasy Mini Kit and RiboPure Yeast Kit), as described above. Cq values that were obtained after RT-qPCR are shown in Table 2. It can be concluded that all RNA extraction methods tested here are valid to perform efficient RT-qPCR, whereby the most efficient amplification of the ITS2 RNA was achieved after RNA extraction with the RiboPure Yeast Kit, in agreement with the abovementioned higher RNA yield with the RiboPure Yeast Kit.

**Table 2:** Cq values obtained for 10^7^ *C. albicans* cells after RT-qPCR of the ITS2 RNA^a^

RNA extraction method	Storage medium	
	TSB-G^b^	RNAlater
RNeasy Mini Kit	12.02 ± 0.74	11.63 ± 0.18
RiboPure Yeast Kit	10.31 ± 0.41	10.48 ± 0.24
NucliSENS easyMAG	11.91 ± 0.51	11.62 ± 0.19

^a^Results are means and standard deviations of six independent extractions

^b^Tryptic Soy Broth + 15% glycerol

## Discussion

In this study, different methods to lyse yeast cells were compared: bead beating with different lysis buffers, vortexing without beads, bead beating with vertical and horizontal orientation of the tubes, lyticase treatment, and cell lysis after storage in plain broth (TSB-G) vs storage in RNAlater. We found that RPLB combined with 10 min of horizontal bead beating with 0.5 mm zirconium beads (as indicated in the RiboPure Yeast Kit’s instruction) led to 100% cell lysis, and therefore this method was selected for further analysis. The presence of RPLB was essential, as we found that horizontal bead beating in saline, resulted in only 48% cell lysis. A comparable result has been reported for *Saccharomyces cerevisiae*, i.e., 57% cell lysis after bead beating in saline, but using glass beads for a longer period, i.e., 30 min [13]. In addition, we also showed that vortexing without beads in RPLB is not sufficient to lyse efficiently yeast cells and demonstrated the importance of using mechanical cell wall disruption in combination with a chemical lysis agent. Several groups studied rapid methods to lyse yeast cells through bead beating, but they did not provide data regarding cell lysis efficiency [16, 17]. We also included lyticase treatment to evaluate cell lysis, but only as a control method (gold standard), because enzymatic methods are not an appropriate choice to lyse yeast cells with the purpose of transcription studies, since they require incubation time during which gene expression profiles may change and during which there may occur RNA degradation, given that the mRNA half-lives range from 4 to 168 min in *C. albicans* [18]. In fact, we established that horizontal bead beating in RPLB offered superior lysis to that obtained with lyticase.

Furthermore, cell lysis was also tested after RNAlater storage. RNAlater is an aqueous solution that stabilizes RNA, preventing its degradation as well as avoiding changes in transcriptomic profiles. The main advantage of RNAlater is that it allows to store samples at room temperature for several hours or at 4 °C for up to one week, according to manufacturer’s instructions. This is especially useful when freezing samples in liquid nitrogen is not possible, as is often the case in a clinical environment. However, according to the Qiagen protocol designated “Optimal RNAlater incubation and removal conditions prior to isolation of total RNA from stabilized cell sample”, it is known that tissues after storage in RNAlater become harder and that greater centrifugal forces are required to pellet cells because of the high density of RNAlater. Therefore, we assessed whether RNAlater might impair cell lysis and actually observed that storage in RNAlater reduced cell lysis in RPLB to 73.5% of the cell lysis efficiency obtained after storage in TSB-G.

After assessing the optimal method for cell lysis after storage in RNAlater, we also compared RNA extraction efficiency for three methods: the NucliSENS easyMAG, the RNeasy Mini Kit and the RiboPure Yeast Kit from samples lysed with easyMAG lysis buffer, RLT buffer + 143 mM β-mercaptoethanol and RPLB, respectively. Since QIAzol Lysis Reagent is also used in combination with the RNeasy Mini Kit, it was omitted from the RNA extraction comparison experiments. We found that the RiboPure Yeast Kit was the most efficient regarding RNA yield. As this kit uses RPLB, this result was not unexpected since the total RNA yield harvested is proportional to the degree of cell lysis, that had been shown to be the highest in RPLB [13]. Furthermore, the three methods also differ in other aspects: the NucliSENS easyMAG extraction is based on magnetic silica particles that capture nucleic acids, while the RNeasy Mini Kit is based on silica spin columns and the RiboPure Yeast Kit is based on phenol:chloroform extraction. Our findings are in line with previous reports that phenol chloroform based RNA extractions yield more RNA than silica based methods [7, 19], although the latter have the advantage that they do not involve the use of hazardous and toxic chemicals such as phenol. Another kit that is based on silica columns, i.e., the NucleoSpin RNA II Kit (Macherey-Nagel, Düren, Germany), has been reported to yield 25–30 μg RNA, starting from 10^8^ cells of *S. cerevisiae* [16]. This result is largely in agreement with the RNeasy Mini Kit with which we obtained 2.8 μg RNA from 10^7^ cells of *C. albicans*. In addition, a recent study, that used formamide-EDTA for RNA extraction from *S. cerevisiae*, obtained an RNA yield comparable to that obtained in this study with the RiboPure Yeast Kit, i.e., 22–30 μg RNA from 3 × 10^7^ cells/ml [20].

Although Nwokeoji et al. [21] reported a yield of 99.57 μg RNA from 10^7^ cells of *S. cerevisiae* with silica-membrane columns (Qiagen or Invitrogen), we only obtained 2.89 μg NA from 10^7^ cells of *C. albicans*, not previously frozen in TSB-G or RNAlater, when we applied their method (Additional file 2: Table S1), using Qiagen silica columns. This result dropped to 2.70 μg after DNase treatment, which is in agreement with our yield obtained with the RNeasy Mini Kit, which uses the same columns, i.e., 2.68 μg after storage at min 80 °C in TSB-G or 2.43 μg after storage at min 80 °C in RNAlater. A brief comparison showing the most relevant differences between these two methods is shown in Additional file 3: Table S2.

Whereas all of these studies were performed in *S. cerevisiae*, studies analyzing RNA yield in *C. albicans* are still lacking. Although several groups have extracted RNA from *C. albicans*, for example, to study differently expressed genes by RNA-seq [5, 6], they did not report on the RNA yield.

Although we assessed that storage in RNAlater reduced cell lysis compared to storage in TSB-G, we observed that, when lysing cells with horizontal bead beating and subsequent extraction of RNA with the RiboPure Yeast Kit, storage in RNAlater did not decrease but increased RNA yield as compared to storage in TSB-G. To the contrary, RNA yield was reduced after storage in RNAlater when RNA was extracted with the RNeasy Mini Kit. Hence, although all extraction methods succeeded to yield RNA after storage in TSB-G and RNAlater, the RiboPure Yeast Kit resulted in (mostly significantly) higher yields (as measured with NanoDrop, Fragment Analyzer, or RT-qPCR) compared to both other methods. However, when RNA integrity is considered, RNeasy Mini Kit and easyMAG showed better quality in samples stored in RNAlater.

## Conclusions

Overall, although all three RNA extraction methods tested here provided high quality RNA, whether or not samples had been stored in RNAlater, we found that the most efficient cell lysis and the highest RNA yield from *C. albicans* cells were obtained with the RiboPure Yeast RNA Extraction Protocol. This is especially relevant in samples that are strongly biased against yeasts, e.g., in infected hosts for which the RNA content from *Candida* constitutes less than 1% of the total RNA content. However, for other purposes, it may be more convenient to sacrifice RNA yield, for example in in vitro experiments in which RNA amounts can be raised simply by using larger amount of cells.

## Methods

### Biological material and growth conditions

*Candida albicans* reference strain ATCC 90028 was grown on Sabouraud Glucose Agar plates with Chloramphenicol (50 μg/ml) (Sigma-Aldrich) and transferred to Yeast extract - Peptone Dextrose broth (Sigma-Aldrich). Cell suspensions were subcultured at 32 °C in static conditions overnight until the logarithmic growth phase was reached. Cells were counted in the microscope by using a hemocytometer (Bürker chamber), adjusted to 10^7^ cells/ml, centrifuged at 8000 *g* for 10 min and pellets were resuspended in 1-ml aliquots in TSB (Becton, Dickinson and Company, Franklin Lakes, NJ) + 15% glycerol (Sigma-Aldrich, St. Louis, MO) and stored at − 80 °C.

### RNAlater treatment

*C. albicans* cells from logarithmic growth phase were harvested by centrifugation during 10 min at 8000 *g*, adjusted to 10^7^ cells/ml with a hemocytometer, resuspended in 1-ml aliquots in RNAlater (Invitrogen, Carlsbad, CA), stored overnight at 4 °C to enable the solvent to penetrate into the cells, and subsequently stored at − 80 °C.

### Bead beating

A total of 24 × 1-ml aliquots of yeast cells in TSB-G and another 24 in RNAlater, that had all been stored at − 80 °C, were thawed, centrifuged during 10 min at 20000 *g* and resuspended in 1-ml aliquots of each of four different lysis buffers: easyMAG Lysis Buffer (bioMérieux, Marcy-l’Étoile, France), RLT Buffer (Qiagen, Hilden, Germany) + 143 mM β-mercaptoethanol (Sigma-Aldrich), QIAzol Lysis Reagent (Qiagen), and RPLB (Ambion, Foster City, CA). All 1-ml aliquots were transferred to prefilled tubes with 0.5 mm zirconium beads and further bead beaten for 10 min. Two different bead mills were compared, a manual vortex mixer (VWR, Radnor, PA) (vertical bead beating) and a hands-free vortex genie-2 (MO BIO Laboratories, Carlsbad, CA), fitted with a vortex adapter, holding microfuge tubes in a horizontal position (horizontal bead beating).

### Lyticase treatment

A total of six 1-ml aliquots, containing 10^7^ yeast cells in TSB-G and another six in RNAlater, all stored at − 80 °C, were thawed, centrifuged during 10 min at 20000 *g*, resuspended in 250 μl of Lyticase Lysis Buffer, i.e., a lysis buffer (50 mM Tris-HCl pH 8.0, 10 mM EDTA, 1.2 M sorbitol, 10 mM β-mercaptoethanol containing 2 U of lyticase/μl (Sigma-Aldrich)) and incubated for 1 h at 37 °C [22], in order to disrupt cell walls from yeast cells to facilitate cell lysis.

### Microscopic visualization and cell viability determination

After the different cell lysis treatments, the cell suspensions were centrifuged for 10 min at 8000 *g* and the pellets were resuspended in 1 ml of saline. Ten μl of this saline cell suspension was loaded into a hemocytometer. Yeast cells could be observed with light microscopy and were counted in 25 small squares (together representing a 0.1 μl volume) at a magnification of 400x. The resulting average number of cells/square was used to calculate the total cell number/ml, as follows: cells/ml = average cell number/square × 2 (dilution) × 10^4^.

### RNA extraction

RNA from lysates after the cell lysis procedure, as optimized in preceding experiments, was isolated using the RNA extraction method of the cognate lysis buffer that had been used: the NucliSENS easyMAG semi-automated extraction platform was used for samples lysed with the easyMAG Lysis Buffer, the RNeasy Mini Kit was used for cells lysed with RLT buffer + 143 mM *β*-mercaptoethanol, and the RiboPure Yeast Kit was used for cells lysed with RPLB, according to manufacturer’s instructions. All nucleic acid extracts were treated with 8 units of DNase I (Invitrogen) for 1 h at 37 °C, in order to remove contaminating chromosomal DNA. Resulting RNA was analyzed for quantity and quality with a NanoDrop spectrophotometer ND-1000 (Isogen Life Science, Utrecht, The Netherlands) and a Fragment Analyzer (Advanced Analytical Technologies Inc., Ankeny, IA, USA).

### RT-qPCR analysis

Reverse transcription was performed for 5 min at 25 °C followed by 60 min at 42 °C with random hexamer primers and with 0.5 μg of total RNA according to the instructions of the manufacturer of the RevertAid First Strand cDNA Synthesis Kit (Thermo Fisher Scientific, Waltham, MA). qPCR was performed in duplicate with a LightCycler 480 (Roche, Basel, Switzerland) to quantify the ITS-2 region as described previously [21, 22]. Universal fungal primers ITS4 (5′-TCC TCC GCT TAT TGA TAT GC-3′) and ITS86 (5′ -GTG AAT CAT CGA ATC TTT GAA C-3′) were used at concentrations of 0.5 mM in the LC480 high resolution melting mix (Roche) and 2 μl of each cDNA product in a final volume of 10 μl. The thermal cycling program consisted of a pre-incubation step for 10 min at 95 °C, amplification for 45 cycles of 20 s at 95 °C, 30 s at 55 °C and 30 s at 72 °C. Results were analyzed with the LightCycler 480 software 1.5 (Roche).

### Statistical analysis

For statistical comparisons of cell lysis data, independent experiments performed with six replicates were considered. Data were analyzed using the statistical test Friedman and the statistical test Wilcoxon for paired samples with IBM SPSS Statistics v 25.0 (IBM, Armonk, NY, USA).

## Additional files

Additional file 1: **Figure S1.** (TIF). Fragment Analyzer electropherograms of total RNA obtained with different RNA extraction methods from 10^7^ *Candida* cells, stored in TSB-G and RNAlater. (TIF 160 kb)

Additional file 2: **Table S1.** RNA yield and quality for different RNA extraction methods^a^. (PDF 178 kb)

Additional file 3: **Table S2.** Comparison between RNeasy Mini Kit and RNASwift RNA purification. (PDF 176 kb)

## Abbreviations

ITS-2: Internal transcribed spacer 2
LLB: Lyticase Lysis Buffer
RPLB: RiboPure Lysis Buffer
TSB-G: Tryptic soy broth + 15% glycerol
