# Supplementary figures and images for: Comparison of the efficiency of different cell lysis methods and different commercial methods for RNA extraction from Candida albicans stored in RNAlater
Source: BMC Microbiol. 2019 May 14;19:94. doi: 10.1186/s12866-019-1473-z (PMC6515685; doi:10.1186/s12866-019-1473-z)

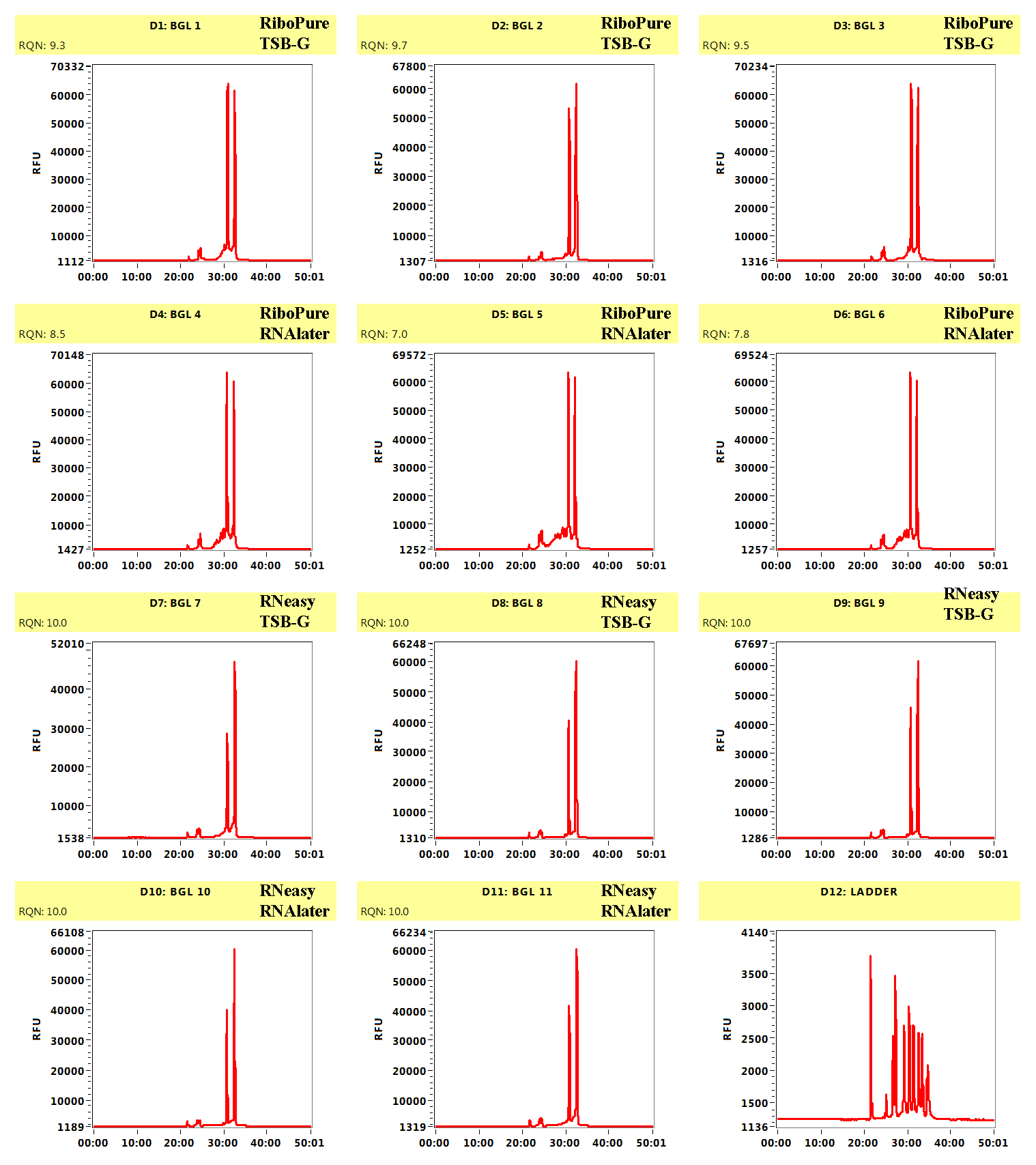

Supplement: Supplementary file 1 — Figure S1. (TIF). Fragment Analyzer electropherograms of total RNA obtained with different RNA extraction methods from 107 Candida cells, stored in TSB-G and RNAlater. (TIF 160 kb) [file 12866_2019_1473_MOESM1_ESM.tif]
