# Supplementary material for: Comparison of the efficiency of different cell lysis methods and different commercial methods for RNA extraction from Candida albicans stored in RNAlater
Source: BMC Microbiol. 2019 May 14;19:94. doi: 10.1186/s12866-019-1473-z (PMC6515685; doi:10.1186/s12866-019-1473-z)
Supplement: Supplementary file 2 — Table S1. RNA yield and quality for different RNA extraction methodsa. (PDF 178 kb) [file 12866_2019_1473_MOESM2_ESM.pdf]

Additional file 2: Table S1. RNA yield and quality for different RNA extraction methods[a]

Method	Storage	RNA (µg/ml)	A260/A280[d]
RiboPure Yeast Kit	TSB-G[b]	6.01 ± 1.27	2.02 ± 0.03
RiboPure Yeast Kit	RNL[c]	7.10 ± 0.22	1.98 ± 0.02
NucliSENS easyMAG	TSB-G[b]	1.86 ± 0.60	2.14 ± 0.08
NucliSENS easyMAG	RNL[c]	2.32 ± 0.20	2.01 ± 0.07
RNeasy Mini Kit	TSB-G[b]	2.68 ± 0.04	1.93 ± 0.04
RNeasy Mini Kit	RNL[c]	2.43 ± 0.11	1.84 ± 0.06
RNASwift	No	2.70 ± 0.27	2.05 ± 0.08

a: Results are means and standard deviations of six independent extractions.

b: Tryptic Soy Broth + 15% glycerol.

c: RNAlater

d: Quality for NanoDrop is shown as the A260/A280 ratio. A value of ~2.0 is generally accepted as indicating that RNA is free of proteins.
