# Supplementary material for: Comparison of the efficiency of different cell lysis methods and different commercial methods for RNA extraction from Candida albicans stored in RNAlater
Source: BMC Microbiol. 2019 May 14;19:94. doi: 10.1186/s12866-019-1473-z (PMC6515685; doi:10.1186/s12866-019-1473-z)
Supplement: Supplementary file 3 — Table S2. Comparison between RNeasy Mini Kit and RNASwift RNA purification. (PDF 176 kb) [file 12866_2019_1473_MOESM3_ESM.pdf]

Additional file 3: Table S2. Comparison between RNeasy Mini Kit and RNASwift RNA purification.

| | RNeasy Mini Kit | RNASwift |
|---|---|---|
| Cell lysis method | Bead beating in RLT+BME | SDS + 0.5 M NaCl at 90 ºC |
| Alcohol added prior purification | 70% ethanol | 60% isopropanol |
| Washing step 1 | RW1[a*] | 15 mM Tris-HCl, 85% ethanol |
| Washing step 2 | RPE[b*] | |
| Washing step 3 | RPE[b*] | |
| DNase treatment | Yes | No |

a: RW1 buffer from Qiagen, containing small amount of guanidine thyocianate and ethanol.

b: RPE buffer from Qiagen, containing ethanol.

*: Buffer composition is confidential.
